# RNAm expression profile of cancer marker genes in HepG2 cells treated with different concentrations of a new indolin-3-one from *Pseudomonas aeruginosa*

**DOI:** 10.1038/s41598-018-30893-w

**Published:** 2018-08-24

**Authors:** Lucas Milanez Benicio, Ane Stefano Simionato, Cláudio Roberto Novello, Jeconias Rocha Guimarães, Ingrid Felicidade, Admilton Gonçalves de Oliveira, João Carlos Palazzo de Mello, Mário Sérgio Mantovani, Andreas Lazaros Chryssafidis, Galdino Andrade, Ilce Mara de Syllos Colus, Marcelo Tempesta de Oliveira

**Affiliations:** 1 Laboratory of Toxicological Genetics, Department of General Biology, State University of Londrina, Londrina, PR Brazil; 2 Microbial Ecology Laboratory, Department of Microbiology, State University of Londrina, Londrina, PR Brazil; 3 Department of General Biology, State University of Londrina, Londrina, PR Brazil; 4 Chemistry and Biology Academic Department, Federal University of Technology-Paraná, Francisco Beltrão, PR Brazil; 5 Pharmaceutical Biology Laboratory, Palafito, State University of Maringá, Maringá, BR- 87020-900 Brazil; 6 Laboratory of Veterinary Toxicology, Department of Preventive Veterinary Medicine, State University of Londrina, Londrina, PR Brazil; 7 Physics and Mathematics Academic Department, Federal University of Technology-Paraná, Francisco Beltrão, PR Brazil

## Abstract

The present study tested the effects of a newly identified indolin-3-one compound (compound **1**), produced by *Pseudomonas aeruginosa*, on HepG2 cells. The MTT assays demonstrated decreased metabolic activities in HepG2 cells treated with compound **1**, with dose- and time-dependent intensifying effect, starting at a concentration of 40 µM. The IC_50_ after 24, 48, 72, and 96 h treatments were 41.35, 52.7, 92.79 and 66.65 μM of compound **1**, respectively. Below 80 µM, no significative damage on erythrocytes membranes was observed by the hemolytic assays. The RT-qPCR revealed that the compound modulated key genes involved in carcinogenesis process, indicating possible indolin-3-one mechanisms of action. The data showed that gene expression alterations promoted by compound **1**, in concentrations up to 60 μM after 48 h, led to a decrease in cellular progression and there was no direct cellular damage. In addition, non-cytotoxic concentrations of compound **1** halved the concentration of the chemotherapeutic doxorubicin, maintaining similar therapeutic effect against HepG2 cells. The novelty of the molecule and the biological activities observed in the present study emphasize the potential of the compound **1** in cancer therapy research.

## Introduction

The indolinone term represents a family of compounds with valuable pharmacological activities, in different therapeutic areas, especially in antineoplastic activity, acting on abnormal cell proliferation, generally by causing protein kinase disorders^1–3^. Indolinones can act directly on tumor cells by selectively blocking tyrosine kinase receptors, proteins responsible for the proliferation and survival of tumor cells, or indirectly by blocking angiogenesis^1–5^. Moreover, these molecules can inhibit cyclin-dependent kinases, proteins involved in the processes of cell cycle control, transcription, cell differentiation, cell death and others^1–3^. Protein kinases play an important role in signal transduction, a strictly regulated cellular process. These proteins modulate cellular responses to external stimuli and influence a number of biological processes, such as cell migration, metabolism, proliferation, survival, differentiation and cycle^3^. Different indolinones have been studied in order to find alternatives to inhibit protein kinases associated with abnormal cell activities, most of them synthesized and some molecules already with patented technologies^1^. The action of indolinone derivatives has been described on MET, VEGF, FGF, FGF, PDGF, c-Kit receptors and others, thus with a potential multi-kinase inhibition effect, acting on several receptors simultaneously^6–10^. The main objective with all the anticancer molecules is to impair tumor cells without consequences for healthy cells, being effective with minimal side effects, in order to control the disease without affecting the quality of life of the patients. This feature challenges research in general, as cancer cells use various strategies for survival, growth and dissemination throughout the body^11^. Despite the different approaches on cancer therapy and prevention, this disease remains one of the leading causes of death worldwide, making necessary the development of new drugs and therapies. The indolinones are pharmacologically important molecules, exhibiting promising and already proven effects in the treatment of cancer and other diseases. Thus, this study aimed on the characterization and chemical definition and identity of a new indolinone compound, produced in the secondary metabolism of *Pseudomonas aeruginosa*, as well as to evaluate the anticancer potential of this molecule.

## Results

### Isolation and Structure Elucidation

**Figure 1:**
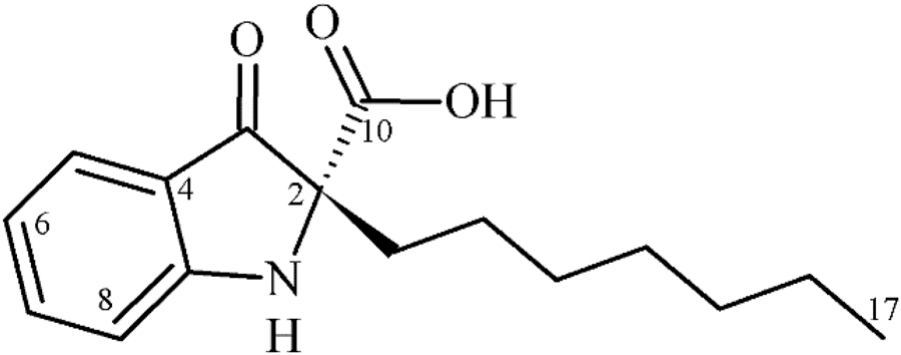
Chemical structure of isolated and identified compound **1**.

The compound **1** was obtained by purification of a metabolite broth used in the fermentation process of *Pseudomonas aeruginosa* LV wild-type strain, isolated from orange plant leaves. The structure and absolute configuration of (*R*)-(+)-2-heptyl-3-oxoindole-2-carboxylic acid was proposed for compound **1** (Fig. 1) based on extensive spectroscopy including 2D NMR spectroscopy, electronic CD (ECD) calculation, and by high resolution mass spectrometry.

Compound **1** was assumed as an off-white amorphous powder, [α]_D_ + 10.0 (C = 0.10, MeOH). The molecular formula was determined as C_16_H_21_NO_3_ by the HR-ESI-MS ion peak at *m/z* 298.1450 [M + Na]^+^, in positive mode (Calculated for C_16_H_21_NO_3_Na, 298,1408). The NMR spectrum presented signals for indol-3-one moiety and *n*–alkyl and carboxyl groups were confirmed by ^1^H- and ^13^C-NMR data assignments. In the ^13^C-NMR spectra, the most significant information was the appearance of signals at δc 195.8 and 174.1 ppm, indicating the presence of the carbonyl function of the 3-oxindole moiety and carboxyl group, respectively. In addition, the quaternary carbon signals at δc 82.9, 119.1, and 140.4, as well as the signal of four sp^2^-methine signals δ_H/C_ 7.87 (dd, *J* 8.1; 1.2 Hz, H-5)/128.0 (C-5), 7.15 (t, *J* 7.5 Hz, H-6)/124.2 (C-6), 7,54 *(*ddd*, J* 8,1; 8.1; 1,2 Hz, H-7)/136.7 (C-7), and 7.10 (d, *J* 8.1 Hz, H-8)/117.0 (C-8), attributed to the aromatic ring, evidencing the presence of the indol-3-one moiety substituted at C-2 position. The *n*-heptyl group was evidenced by methylic carbon signal at δ_H/C_ 0.79 (t, *J* 6.9 Hz, H-17)/14.2 (C-17), together with five methylenic signals at δ_H/C_ 1.80 (ddd, *J* 13.8; 11.4; 5.1 Hz, H-11)/41.2 (C-11), 1.35 (m, H-12)/22.9 and 1,16 (m, H-13 to H-16)/29.1, 29.4, 31.8 and 22.7 (C-13 to C-16) ppm. The correlations presented in ^1^H/^1^H-COSY are consistent with the proposed assignments. Correct positioning of the carbonyl, carboxyl and *n*-heptyl groups were verified through HMBC experiment. The correlations of the carbonyl δ_C_ 195.8 and carboxyl δ_C_ 174.1 groups with the methylenic hydrogens in δ_H_ 1.93 (H-11a) and 1.80 (H-11b) confirmed the assignment of these groups in C-1 and C-10 position, respectively. The confirmations that heptyl group is attached to the C-2 quaternary carbon was made by the correlation of the methylenic hydrogens in δ 1.93 (H-11a) and 1.80 (H-11b) with the signal at δ 82.9. The physical and spectral data of the products are given as supporting information (Figures S1–S6).

In order to determine the absolute configuration at C2 of **1**, we used Density Functional Theory (DFT) to obtain geometry ground state and time dependent DFT to calculate its electronic excited states. DFT is the most used method to perform molecular quantum calculation, given its satisfactory results for geometry and electronic states and low computational cost. Its most notable characteristic is the electronic density as main variable, but it also yields exact ground state of a multi electronic systems, as ensured by the two theorems in which is based^12,13^. For the analyzed systems, first we calculated the lowest energy conformation and then the electronic excited states were obtained. We chose functional B3LYP and basis set 6–31 G** as implemented on Gaussian 09 package.

**Figure 2:**
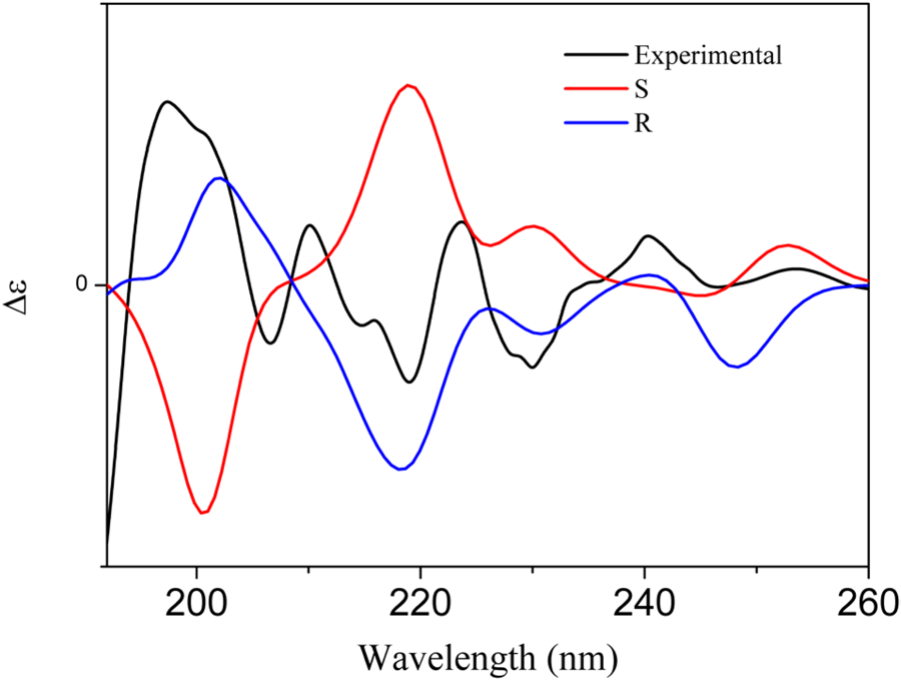
CD and ECD spectra for compound **1**.

For both compounds (*R* and *S* indolinone), we tested some conformational features: (i) hydrogen bonded to nitrogen and (ii) the torsion of carboxylic group. The final geometry has hydrogen pointing towards the oxygen on the carbonyl. Finally, with the structural lowest energy, excited states were calculated and a theoretical circular dichroism spectra (CD) was obtained following the equation shown on the reference^14^. The results showed that the ECD spectra of **1** shared the almost identical Cotton effect with the calculated CD for *R*, but opposite to the calculated CD of *S* indolinone structure, respectively (Fig. 2). The structural absolute configurations of **1** was established with *R*.

### Cytotoxic screening of Indolin-3-one (compound 1) on HepG2 cells and human erythrocytes

**Figure 3:**
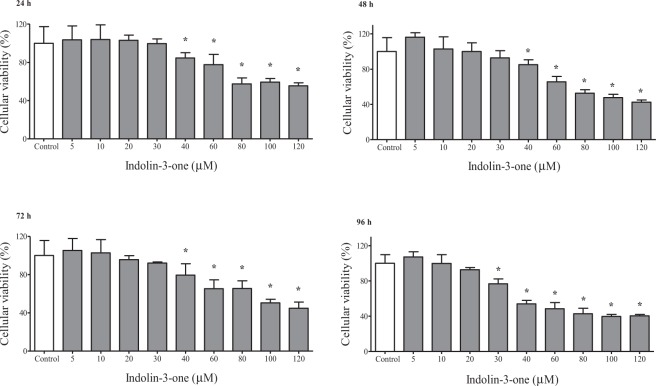
MTT assay for cellular viability evaluation of HepG2 cells treated with 5, 10, 20, 30, 40, 60, 80, 100 and 120 µM of indolin-3-one in 24, 48, 72 and 96 hours. The results are expressed as percentages relative to control. *Indicates statistically significant differences in relation to the respective control means compared by ANOVA followed by Dunnet’s test (*p* < 0.05), using the GraphPad Prism 5 software.

**Figure 4:**
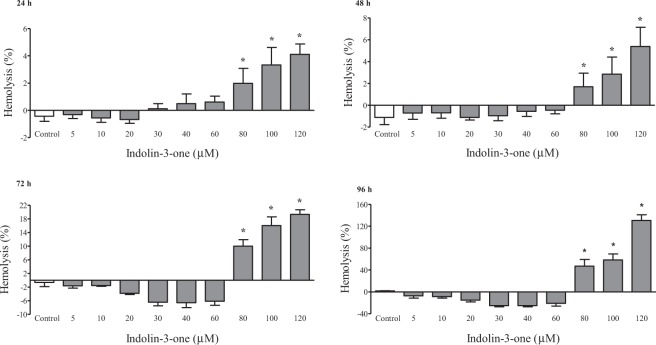
Hemolytic assay for membrane integrity evaluation of erythrocytes treated with 5, 10, 20, 30, 40, 60, 80, 100 and 120 µM of indolin-3-one in 24, 48, 72 and 96 hours. The results were expressed as percentage of hemolysis by the equation: (Treatment absorbance − negative control absorbance)/(positive control absorbance − negative control absorbance) × 100. *Indicates statistically significant differences in relation to the respective control means compared by ANOVA followed by Dunnet’s test (*p* < 0.05), using the GraphPad Prism 5 software.

To assess the cytotoxic effects of indolin-3-one, initially the MTT [3-(4,5-dimethylthiazol-2-yl)-2,5-diphenyltetrazolium bromide] assays were carried out using HepG2 cells treated with different concentrations of compound **1**, at four distinct incubation times (Fig. 3). At 24, 48, and 72 h of incubation, indolin-3-one significantly decreased the proliferation of HepG2 cells from the concentration of 40 μM, and at 96 h from 30 μM. This effect intensified in a dose- and time-dependent manner. The polynomial regression analysis showed that for each µM increased in the concentration of indolin-3-one, a decrease of 0.48%, 0.60%, 1.27% and 0.56% in cellular proliferation occurred, respectively at 24, 48, 72, and 96 h of treatment. The calculated IC_50_, based on absorbance values, for 24, 48, 72, and 96 h of treatment were, respectively, 41.35, 52.7, 92.79 and 66.65 μM of indolin-3-one. Additionally, a hemolytic assay was performed to evaluate the sensibility of human erythrocytes to indolin-3-one, using the same experimental conditions of MTT assays (Fig. 4). This experiment was an additional test to confirm and select the concentrations of indolin-3-one that do not cause direct membrane damages and cellular death on healthy cells. Concentrations up to 60 μM of indolin-3-one did not cause physical damage to the erythrocyte membrane at any time of exposure, with significant hemolytic effect only observed at concentrations of 80 μM and above. It is worth mentioning that the percentage of hemolysis did not exceed 10% for all concentrations tested in 24 and 48 h, a parameter usually applied to indicate significantly cytotoxicity^15^.

### Associated treatments of non-cytotoxic concentrations of indolin-3-one and chemotherapeutic doxorubicin on HepG2 cells

**Figure 5:**
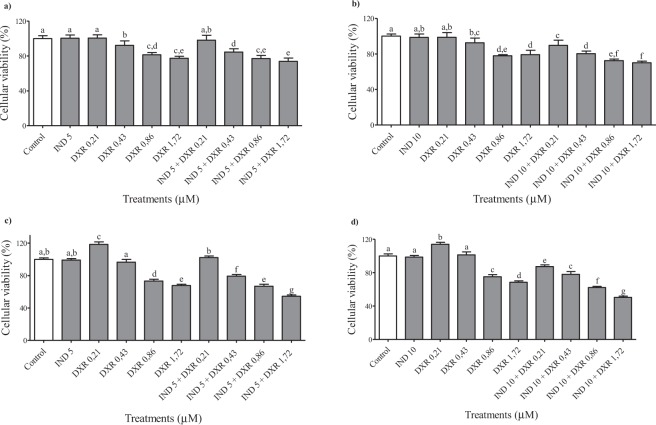
MTT assay for cellular viability evaluation of HepG2 cells treated with non-cytotoxic concentrations of indolin-3-one (IND) associated with 0.21, 0.43, 0.86 and 1,72 µM of chemotherapeutic doxorubicin (DXR). indolin-3-one in 24, 48, 72 and 96 hours. The results are expressed as percentages relative to control. *Indicates statistically differences between the multiple conditions comparison by ANOVA followed by Tukey’s test (*p* < 0.05), using the GraphPad Prism 5 software.

To evaluate the influence of non-cytotoxic concentrations of indolin-3-one on the therapeutic action of doxorubicin, 5 and 10 μM of indolin-3-one were associated with 4 different doses of doxorubicin and incubated for 24 and 48 h (Fig. 5). The association of 5 μM induced a significant increase in the action of doxorubicin at 48 h of treatment. This increase was greater when the concentration of indolin-3-one raised to 10 μM, at both incubation times. At 48 h of treatment, the indolin-3-one at 10 μM allowed to halve the concentration of doxorubicin, while keeping a statistically similar therapeutic effect against HepG2 cells (i.e. 0.43 μM of DXR associated with 10 μM of indolin-3-one). In some conditions, less doxorubicin associated with the new compound promoted better action than twice the doxorubicin dose alone (i.e. 0.21 μM of DXR associated with 10 μM of indolin-3-one).

### RT-qPCR analysis of tumor suppressors and proto-oncogenes of HepG2 cells exposed to non-cytotoxic concentrations of indolin-3-one

**Figure 6:**
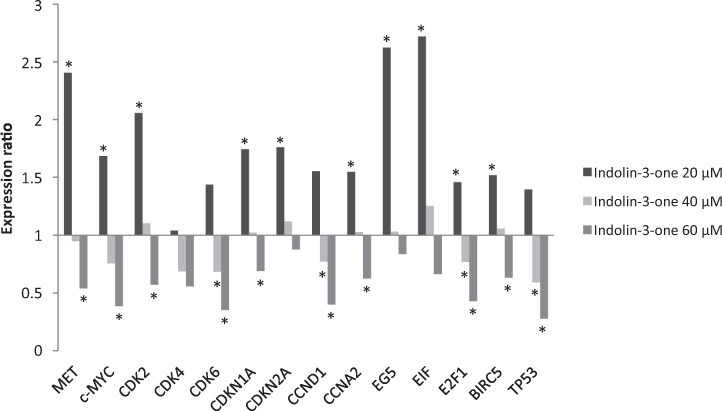
RT-qPCR analysis of cancer related genes in HepG2 cells treated with 20, 40 and 60 µM of indolin-3-one during 24 hours of exposure. *Indicates statistically difference in relative expression compared to the control (*p* < 0.05) using *GAPDH* and *HPRT1* as references genes. Statistical evaluation of reference gene and target expression levels were performed using the standalone software REST 2009, with efficiency correction. All expression levels, standard errors, 95% confidence index and *p* values are described in Table S1.

**Figure 7:**
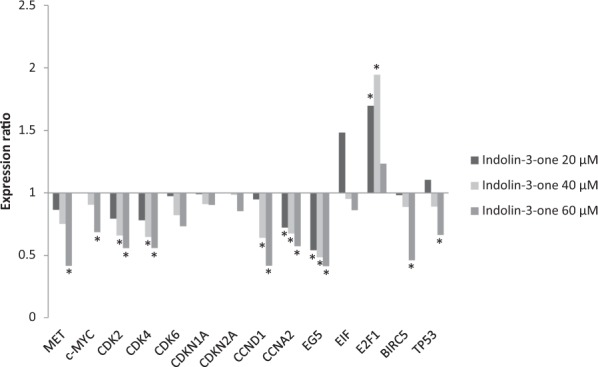
RT-qPCR analysis of cancer related genes in HepG2 cells treated with 20, 40 and 60 µM of indolin-3-one during 48 hours of exposure. *Indicates statistically difference in relative expression compared to the control (*p* < 0.05) using *GAPDH* and *HPRT1* as references genes. Statistical evaluation of reference gene and target expression levels were performed using the standalone software REST 2009, with efficiency correction. All expression levels, standard errors, 95% confidence index and *p* values are described in Table S2.

Due to the various scientific studies reporting the effects of indolinones against cancer, genes involved directly or indirectly in the carcinogenesis process were selected for evaluation of their change in expression after 24 and 48 h of treatment with indolin-3-one: *MET*, *c-MYC*, *CDK2*, *CDK4*, *CDK6*, *CDKN1A*, *CDKN2A*, *CCND1, CCNA2*, EG5 (*KIF11*), *EIF*, *E2F1*, *BIRC5* and *TP53*. As the hemolytic test results indicated that concentrations below 80 μM did not cause direct physical damage to erythrocytes membranes, the concentrations of 20, 40, and 60 μM were selected to assess the influence of indolin-3-one on the modulation of gene expression related to carcinogenesis. Furthermore, previously to mRNA extraction, the viability of HepG2 cells was verified using a Countess Automated Cell Counter (Thermo Fisher Scientific) and Trypan Blue reagent, using cells treated with 20, 40, and 60 μM of indolin-3-one, at 24 and 48 h. In all these conditions, the cell viability was greater than 90%. In general, indolin-3-one significantly modulated the expression of most genes in a dose and time-dependent manner (Figs 6 and 7, Tables S1 and S2). These results were similar to the viability of HepG2 cells evaluated by MTT assays. Within 24 h, the *MET*, *c-MYC*, *CDK2*, *CDKN1A*, *CDKN2A*, *CCNA2*, *EG5*, *EIF, E2F1* and *BIRC5* genes were up-regulated when cells were treated with 20 μM of indolin-3-one. In the same concentration, at 48 h of treatment, this pattern started to invert and almost all gene expression rates returned to normal, except for *CCNA2* and *EG5*, which were down-regulated and *E2F1* that was up-regulated. When concentration increased to 40 μM, at 24 h of treatment, only the *CDK6*, *CCND1*, *E2F1* and *TP53* genes were down-regulated. The other genes remained unchanged. At 48 h of treatment with 40 μM of indolin-3-one, the *CDK2*, *CDK4*, *CCND1*, *CCNA2* and *EG5* genes were down-regulated and only *E2F1* gene was up-regulated. The treatment with 60 μM of indolin-3-one promoted the down-regulation of all genes, except for *CDK4*, *CDKN2A*, *EG5* and *EIF* at 24 h of treatment, and *CDK6*, *CDKN1A*, *CDKN2A*, *EIF* and *E2F1* at 48 h, which maintained a normal expression rate. It is noteworthy that although these genes have not presented significant changes like the others, they presented a tendency of decreasing expression.

## Discussion

In addition to having a natural origin, the first differential evidence of the molecule described in the present study was the indolin-3-one scaffold, since the majority of indolinone compounds developed and marketed for cancer treatment is synthesized from indolin-2-one derivatives^1,2,11,16^. In this context, the compound **1** also can be used as a novel precursor for chemical optimization. An initial assay was performed to evaluate the anticancer potential of this new indolin-3-one. The reduction of the tetrazolium dye (MTT) to formazan by dehydrogenases of viable cells, provides an indication of the metabolic activity, reflecting its potential for growth and survival, interpreted as the percentage of cellular viability. The reduction of MTT does not occur only in mitochondria. The process is NADH and NAD(P)H-dependent and it is also reduced in other cellular compartments, such as the cytoplasm and regions of the plasma membrane^17,18^. So, the MTT assay allow us to make an assumption of the cell energetic state, which may or may not be from cell death or cycle arrest. A decrease in cellular proliferation of HepG2 cells treated with compound **1** was observed, starting at 40 µM, with dose- and time-dependent increasing effect. The hemolysis test, carried out in the same conditions of MTT assay, evidenced that concentrations up to 60 μM of indolin-3-one did not cause physical damage to the erythrocyte membrane at any time of exposure. The spectrophotometric detection of hemoglobin released by erythrocytes membrane injuries is an extremely sensitive technique, being especially important for agents that are also intended to be directly introduced into the bloodstream^19^. Due to its sensibility, this trial allowed an efficient selection of indolin-3-one concentrations that did not cause direct membrane damages, since there was no intention to work with concentrations capable of causing such cytotoxic effect or cell death. In addition, before the gene expression analysis, it was verified by Trypan Blue Exclusion assay that 20, 40 and 60 µM of indolin-3-one did not alter HepG2 cells viability at 24 and 48 h of treatment. These results suggested that the reduction of HepG2 cellular progression in concentrations up to 60 μM of indolin-3-one, was not due to direct physical injuries, or biological processes that induce membrane damages and death, but a consequence of some type of modulation at the genetic level. The gene expression analyses reinforced these findings, once all genes (except *E2F1*) were significantly down-regulated or presented a tendency of decreasing under indolin-3-one at 48 h, in a dose- and time-dependent manner. The *MET* gene has increased expression in a variety of cancers when compared to healthy tissues. Its binding to hepatocyte growth factor (HGF) triggers a cascade of reactions that results in a large number of biological responses, which contribute to tumorigenesis. For this reason, several approaches for blocking *MET* activation are under development, such as tyrosine kinase inhibitor molecules^8,20,21^. The data suggest that indolin-3-one treatment can keep *MET* suppressed and the cell would not become malignant by this activation pathway. There are previous approaches targeting *MET* in clinical practice and in preclinical phases of study, among them the use of antagonistic molecules, antibodies, inhibitors of protein kinases and those that reduce their expression^22,23^, in which the newly described indolin-3-one would fit. As observed to *MET*, indolin-3-one can also suppress *c-MYC* gene expression. *c-MYC* depletion inhibits proliferation of human tumor cells at various stages of the cell cycle, being essential for sustaining proliferation^23–25^. Both *c-MYC* and *MET* are proto-oncogenes related to several cancer hallmarks, like sustaining proliferative signaling, evading growth suppressors, enabling replicative immortality and activating invasion and metastasis^25^. Thus, treatment with indolin-3-one has the potential to act on cancer hallmarks and contribute to a lower cell proliferation, contributing for the non-occurrence of other malignant pathways, interesting for the treatment of tumor cells.

*CCND1*, *CCNA2*, *CDK2*, *CDK4* and *EG5*, part of the main regulatory genes of cell cycle control and progression, also closely linked to cancer hallmarks and their related genes (as *MET* and *c-MYC*)^23–26^, were all down-regulated by indolin-3-one in a dose and time-dependent manner. Other correlated genes, although not significantly changed in 24 h treatment, also presented the same dose- and time-dependent reduction pattern. The evidence that CDKs, their regulators and substrates are targets for genetic alteration in different types of human cancer, has stimulated the search for chemical CDKs inhibitors^27–29^. Again, the compound described in the present study fulfills these goals, and more.

The search process for new anti-cancer agents is entering a new era: it has moved from identifying compounds that directly kill tumor cells towards a more mechanistic strategy, acting on molecular targets that underlie cell transformation^28–30^. Carcinogenesis is a complex event controlled by several signal transduction pathways. The broadly multivariate nature of the molecular-level changes involved in any cancer leads to specific hallmarks profiles, which makes the tumorigenesis a complex and multistep process that involves the accumulation of successive transformational events with multi-factorial etiology. Nevertheless, such events result in the acquisition of key hallmark characteristics that are shared by all cancer cells^11,26^. In this context, a new generation of anticancer agents emerges in order to fulfill this purpose in a more specific and effective way, interfering in the signaling of oncogenic events, intrinsic and extrinsic to the tumor cells and to the tumor microenvironment, without the need of elaborated methodologies for their application^11,27–29^. Among the main targets used for this objective, there are proto-oncogenes, tumor suppressors and tyrosine kinase receptors^11,26–29^. The indolin-3-one carries the characteristics of these new generation anticancer agents, as evidenced by the results of the present work, modulating favorably these target genes, even when non-cytotoxic concentrations are employed.

Beyond that, the association of indolin-3-one with half the therapeutic dose of doxorubicin promoted better action than twice the doxorubicin dose alone. Combined drugs are also less prone to elicit drug resistance, leading to some improved pharmacological properties than each individual compound. As indole nucleus is a central component of many natural and synthetic molecules with extensive biological activity, these compounds recently have been used as a “multi-target approach”, in order to design and develop agents able to act simultaneously on multiple intracellular constituents and signaling pathways, leading to better therapeutic effects^11^. Once more, the indolin-3-one fits these purposes and, together with other molecules, it can be used in the development of hybrid drugs for multi-target cancer therapy, a new approach area to evade cancer drug resistance and to design new agents, built to specifically target intracellular components and signaling pathways in cancer^11^.

In summary, a new indolin-3-one compound that contributes efficiently to this accomplishment was discovered. The identified compound has the necessary characteristics to reach objectives like the simultaneous modulation of more than one biological target for cancer chemotherapy; in combination chemotherapy, it can enable different mechanisms of action, thereby, being more effective, decreasing cancer cell resistance and minimally overlapping toxicity profile; thus reducing side effects and consequently improving patient compliance, among other characteristics. Taken together, these facts justify the continuity of future works for a more detailed characterization of the potentials evidenced in this first study.

## Methods

### General experimental procedures

Optical rotation and circular dichroism was measured on a JASCO P-2000 polarimeter and JASC0 J-815 CD spectrometer in MeOH, respectively. IR spectra were recorded on a Bomem MB-Series spectrometer, in a 100 to 3500 cm^−1^ spectral region, using KBr matrix HR-ESI-MS and spectrum was recorded in a ESI-MS Quattro LCZ (Micromass Manchester, UK). NMR spectra were recorded on a Varian Mercury Plus 300 MHz spectrometer (7.02 T), operated at 75 MHz for ^13^C and 300 MHz for ^1^H and 2D NMR and a Bruker (Avance III) 400 MHz instruments. Column chromatography was carried out in Silica gel (70–230 mesh, Merck) for vacuum liquid chromatography (VLC) and Silica gel C-18 (HF Bondesil C-18, Agilent) for flash chromatography (FC). Thin-layer chromatography (TLC) was performed on precoated silica gel aluminum sheets (Kieselgel 60 F254, 0.20 mm, Merck, Darmstadt, Germany) and visualized in UV 254 or 365 nm.

### Bacterial strain

The bacteria used was *Pseudomonas aeruginosa* LV strain wild-type. This strain was isolated from leaves of orange plants, at Astorga city, Brazil. Glycerol stocks were prepared and stored at −20 °C. The LV strain was deposited in the Microbial Culture Collection of Laboratory of Microbial Ecology, Londrina State University, Brazil.

### Process for metabolites production

The fermentation parameters such as medium, pH, inoculum load, agitation, aeration, temperature and culture age were standardized to optimize the growth of *P. aeruginosa* LV strain and the production of metabolites has been patented (Patent #PI0803350-1; www.inpi.gov.br) and described by de Oliveira *et al*.^31^. The culture was harvested and centrifuged at 9.000 rpm for 15 min at 4 °C. The metabolites were extracted five times from the cell-free culture supernatant using two volumes of dichloromethane each time and was named dichloromethane phase (DP). The extracts were pooled and concentrated by rotary evaporation at 40 °C.

### Purification of metabolites

The dichloromethane phase (DP) (60 mg/L) was purified by VLC, carried out in a glass column (350 × 20 mm) coupled to a vacuum pump with −350 mmHg. The columns were eluted with 18 portions of 200 mL of each following mobile phase: hexane (F1a and F1b), hexane:dichloromethane (1:1, F2a and F2b), dichloromethane (F3a and F3b), dichloromethane:ethyl acetate (1:1, F4a and F4b), ethyl acetate (F5a and F5b), ethyl acetate:methanol (1:1, F6a and F6b), methanol (F7a and F7b), methanol:water (1:1; F8a and F8b) and water (F9a and F9b). The fractions were combined and concentrated in a rotary evaporator (Rotavapor R 215, Büchi) at 45 °C, *in vacuo* monitored by TLC in order to identify the presence of indole compound.

The F4b fraction (20 mgL) (indole compound presence) was purified again by FC. The column (500 × 8 mm) was coupled to a low-pressure pump and eluted using a mobile phase with different proportions of water:acetonitrile [85:15 and 60:40, (v/v)] and acetonitrile [100]. The fractions were collected and monitored by TLC. The similar fractions were combined and six fractions were obtained. One milligram (1 mg) of a pure compound (compound **1**) was obtained and was subjected to chemical characterization.

*R-*(+)*-2-heptyl-3-oxoindole-2-carboxylic acid* (**1**): off-white amorphous powder; [α]_D_^25^ + 10.0° (*c* 0.10, MeOH). CD (MeOH) λ_max_ (Δε) 197 (13.4), 219 (−7,4) nm; IR (KBr) ν_max_ (cm^−1^) 3157, 2596, 1333, 1298, 1105, 999, 919 and 333 cm–1; ^1^H-NMR (300 MHz) δ 7.87 (*dd*, J = 8.1, 1.2 Hz, 1 H, H-5), 7.54 (*ddd*, J = 8.1, 8.1, 1.2 Hz, 1 H, H-7), 7.15 (*t*, J = 7.5 Hz, 1 H, H-6), 7.10 (*d*, 8.1 Hz, 1 H, H-8), 1.93 (*ddd*, J = 13.8; 11.4; 4.8 Hz, 1 H, H-11a), 1.80 (*ddd*, 13.8, 11.4, 5.1 Hz, 1 H, H-11b), 1.35 (*m*, 2 H, H-12), 1.16 (*m*, 4 H, H-13 to 16), 0.79 (*t, J* = 6.9 Hz, 3 H, H-17); ^13^C-NMR (75 MHz) δ 195.8 (C-3), 174.1 (C-10), 140.4 (C-9), 136.7 (C-7), 128.0 (C-5), 124.2 (C-6), 119.1 (C-4), 117.0 (C-8), 82.9 (C-2), 41.2 (C-11), 31.8 (C-15), 29.4 (C-14), 29.1 (C-13), 22.9 (C-12), 22.7 (C-16), 14.2 (C-17); HR-ESI-MS (m/z): [M + Na]^+^ m/z 298.1450 (calcd for C_16_H_21_NO_3_Na, 298.1408).

### Cell Line and Culture Conditions

The hepatocellular carcinoma cell line HepG2 is of great relevance to detect cytotoxic and genotoxic substances. This cell line is a tool for chemical risk assessment and is a well-established cellular model for *in vitro* anti-tumor efficiency assays^32–34^. For this reason, HepG2 cells were chosen to investigate the *in vitro* anti-tumor activity of indolin-3-one and its underlying mechanisms. In addition, the use of HepG2 cells is a good *in vitro* experimental model, for it expresses some inducible phase I and II metabolic enzymes and can predict *in vitro* hepatotoxic effects of drugs in humans^35,36^. HepG2 were kindly provided by the Laboratory of Nutrigenomics of FCFRP of University of São Paulo, Brazil. HepG2 cells were grown in 25 cm^2^ culture flasks with 10 mL of Dulbecco’s Modified Eagle Medium (DMEM – low glucose) supplemented with 15% of fetal bovine serum (FBS) and maintained in an atmosphere of 5% CO_2_ at 37 °C.

### MTT Assay

Cytotoxicity was assessed using the MTT assay, based on the protocol described by Mosmann^37^, with modifications. Cell suspension was seeded at 1.0 × 10^4^ cells per well in 96-well plates containing 200 μL of culture medium supplemented with 15% of FBS. After 24 h of stabilization, the medium of each well was removed and 9 different concentrations of the indolin-3-one (5, 10, 20, 30, 40, 60, 80, 100, and 120 µM solubilized with 1% of methanol in DMEM plus 15% FBS) were added. A solvent control condition (DMEM + 15% FBS + 1% methanol) were also included and used as control to statistical analysis. After each treatment period (24, 48, 72 and 96 h), the culture medium was replaced by 100 µL of MTT (0.5 mg/ml), solubilized in serum-free DMEM and incubated for 4 h. Finally, the MTT solution was removed and the formazan crystals generated were solubilized using 100 µL of DMSO. The absorbance was measured using a Biotek spectrophotometer at 570 nm. The mean absorbance of each reaction was converted to cell viability (%) using the following equation: (mean absorbance treatment/mean absorbance control) × 100.

### Hemolysis assay

The hemolytic activity of indolin-3-one was measured by the hemoglobin release assay according to protocol described by Alves *et al*.^38^, with modifications. Red Blood Cells (RBCs) from of a healthy individual with A^+^ blood typing were used. Six hundred microliters (600 μL) of phosphate buffered saline (PBS) solution plus 4% RBCs were exposed to nine different indolin-3-one concentrations cited above and incubated at 37 °C for 24, 48, 72 and 96 h. Aliquots of 100 μL of the same suspension were withdrawn at each incubation time, after 5 minutes of centrifugation at 1000 rpm. The amount of hemoglobin released was measured in Biotek spectrophotometer at 540 nm. RBCs incubated only in PBS were used as negative control of hemolysis. A positive control for 100% membrane damage was used by treating cells with 1% of Triton – X 100 to PBS solution. The results were expressed as percentage of hemolysis by the equation: (absorbance of the treatment − absorbance of the negative control)/(absorbance of the positive control − absorbance of the negative control) × 100.

### Trypan blue exclusion assay

According to manufacturer’s recommendations, we used the Countess® Automated Cell Counter to determine the cell number and viability of HepG2 cells treated with 20, 40 and 60 μM of indolin-3-one for 24 and 48 h at 37 °C and 5% CO_2_.

### Gene expression analysis using RT-qPCR

Sixty thousand (6 × 10^4^) cells per well were inoculated in a 24-wells plate with 1 mL of DMEM plus 15% FBS for 24 h at 37 °C and 5% CO_2_ for stabilization. Then, the culture medium was replaced by the following treatments: 20, 40 and 60 μM of indolin-3-one in DMEM plus 15% FBS. We used 1% methanol in DMEM plus 15% FBS as control condition. The cultures were incubated for 24 and 48 h. Total RNA was extracted using Trizol Reagent, according to the supplier’s instructions. The purity and concentration of isolated RNA were determined by spectrophotometer and RNA integrity and quality were verified by denaturing agarose gel electrophoresis^39^. Capillary electrophoresis using Bioanalyser 2100 (Agilent Technologies) was carried out to determine the RNA integrity number (RIN) (Figures S7 and S8). cDNA synthesis of each sample was done in triplicate in the Veriti Thermal Cycler (Applied Biosystems) using 250 ng of total RNA, diluted in a final volume of 16 µL, containing oligo dT (80 pmol), random primers (100 pmol) and dNTPs (0.5 mM). This first reaction mix was incubated for 10 min at 65 °C. Each reaction was then thermal shocked on ice and 4 µL of a second reaction mix was added [1.3 µL of DEPC- treated H_2_O, 2 µL of Buffer 10 × , 0.6 µL of MgCl_2_ (50 mM), 0.05 µL of RNase Out (Invitrogen), 0.05 µL of SuperScript III enzyme (Invitrogen)]. The final solutions were submitted to a 37 °C incubation for 50 minutes to allow cDNA synthesis and a final enzyme inactivation at 70 °C for 15 min. The qPCR reactions were performed in triplicate in CFX96^TM^ Real-Time System (Bio-Rad) using 5 µL of SsoAdvancedTM SYBR® Green Supermix (Bio-Rad), 1 µL of each oligonucleotide primer (10 pmol/µL) and 5 µL of cDNA (50 ng/µL) (1:10 dilution of input RNA). Reaction conditions are the following: pre-incubation of 50 °C for 2 min (UDG incubation), initial denaturation at 95 °C for 5 min; and 45 cycles of: 95 °C/20 s, 60 °C/30 s, 72 °C/20 s. A melting curve analysis ranging from 50 °C to 98 °C was performed in the end of the reaction, with 5 s reading in every 0.5 °C. The software CFX Manager 3.1 (Bio-Rad) was used to collect the data and the efficiency of the reactions was calculated by LinRegPCR software (RUIJTER *et al*., 2009; RUIJTER *et al*.)^40,41^. *GAPDH* and *HPRT1* were used as reference genes. Target genes were the following: *MET*, *c-MYC*, *CDK2*, *CDK4*, *CDK6, CDKN1A*, *CDKN2A*, *CCND1*, *CCNA2*, *EG5*, *EIF*, *E2F1*, *BIRC5*, and *TP53*.

### Statistical analysis

The absorbance values obtained from MTT and hemolysis assays were compared by ANOVA followed by Dunnet’s test (*p* < 0.05) using the GraphPad Prism*®* 5. In Indolin-3-one/DXR associated treatments we conducted ANOVA followed by Tukey’s test (p < 0.05). The Software R was used for the polynomial regression analysis and the calculated IC_50_. Statistical validation of reference gene and gene expression levels were determined by the stand-alone software REST® 2009 (*Relative Expression Software Tool*/Qiagen®)^42^. Statistical difference was defined as a 2-fold change variation plus a *p* value < 0.05, when each treatment was compared to the control, as well as fold change variations with *p* values of p ≤ 0.01.

## Electronic supplementary material

Supplementary Figures

Supplementary Tables
