# Supplementary material for: RNAm expression profile of cancer marker genes in HepG2 cells treated with different concentrations of a new indolin-3-one from Pseudomonas aeruginosa
Source: Sci Rep. 2018 Aug 24;8:12781. doi: 10.1038/s41598-018-30893-w (PMC6109079; doi:10.1038/s41598-018-30893-w)
Supplement: Supplementary file 1 — Supplementary Figures [file 41598_2018_30893_MOESM1_ESM.pdf]

Supplementary Figures

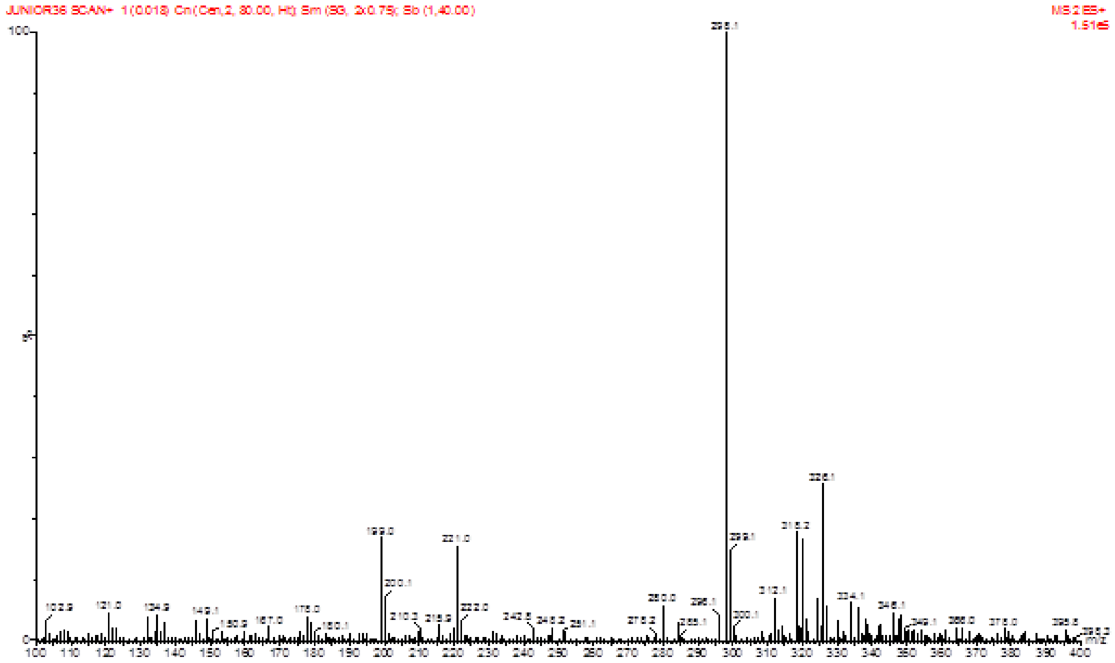

Figure S1. ESI-MS spectrum of compound **1** (negative mode).

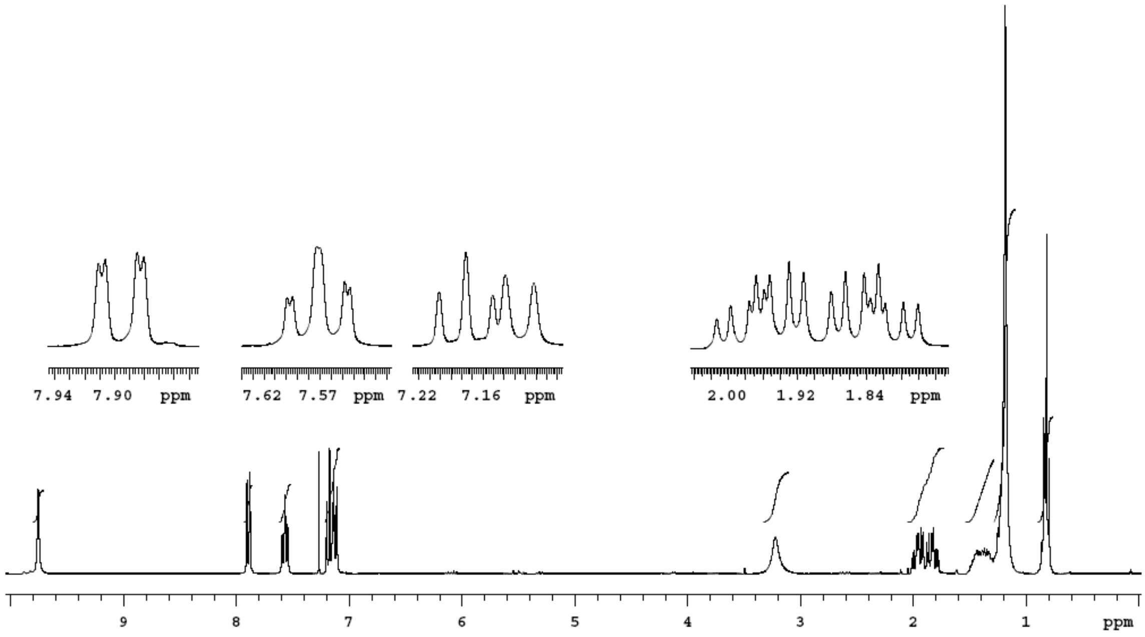

Figure S2. ^{1}H NMR spectrum of compound **1** (300 MHz, CD_3OD).

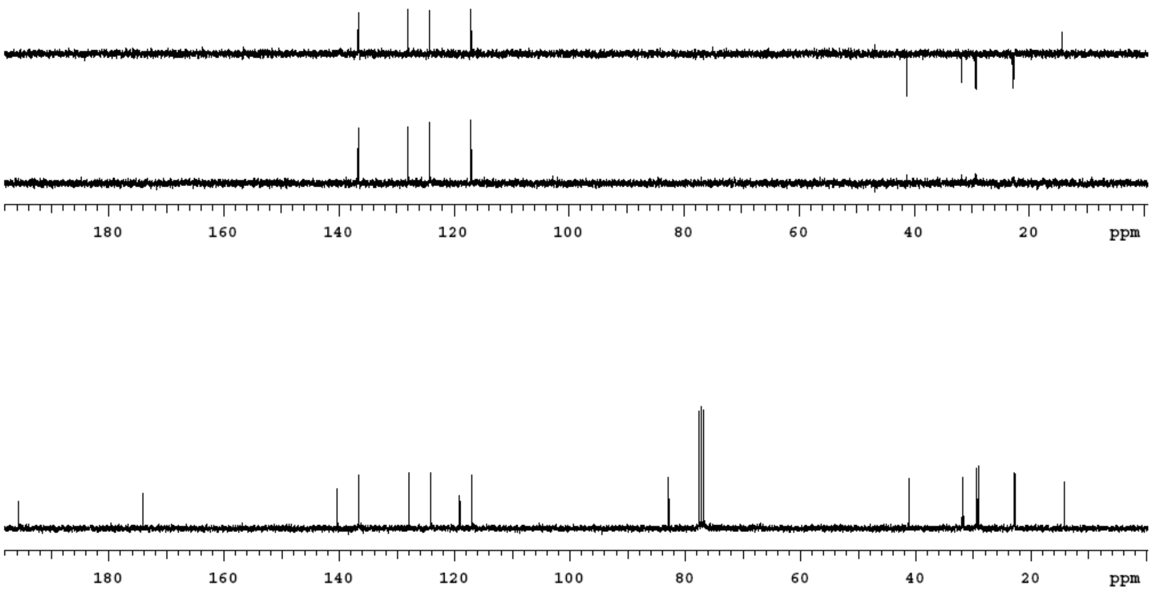

Figure S3. DEPT spectrum of the compound **1** (300 MHz, CD_3OD)

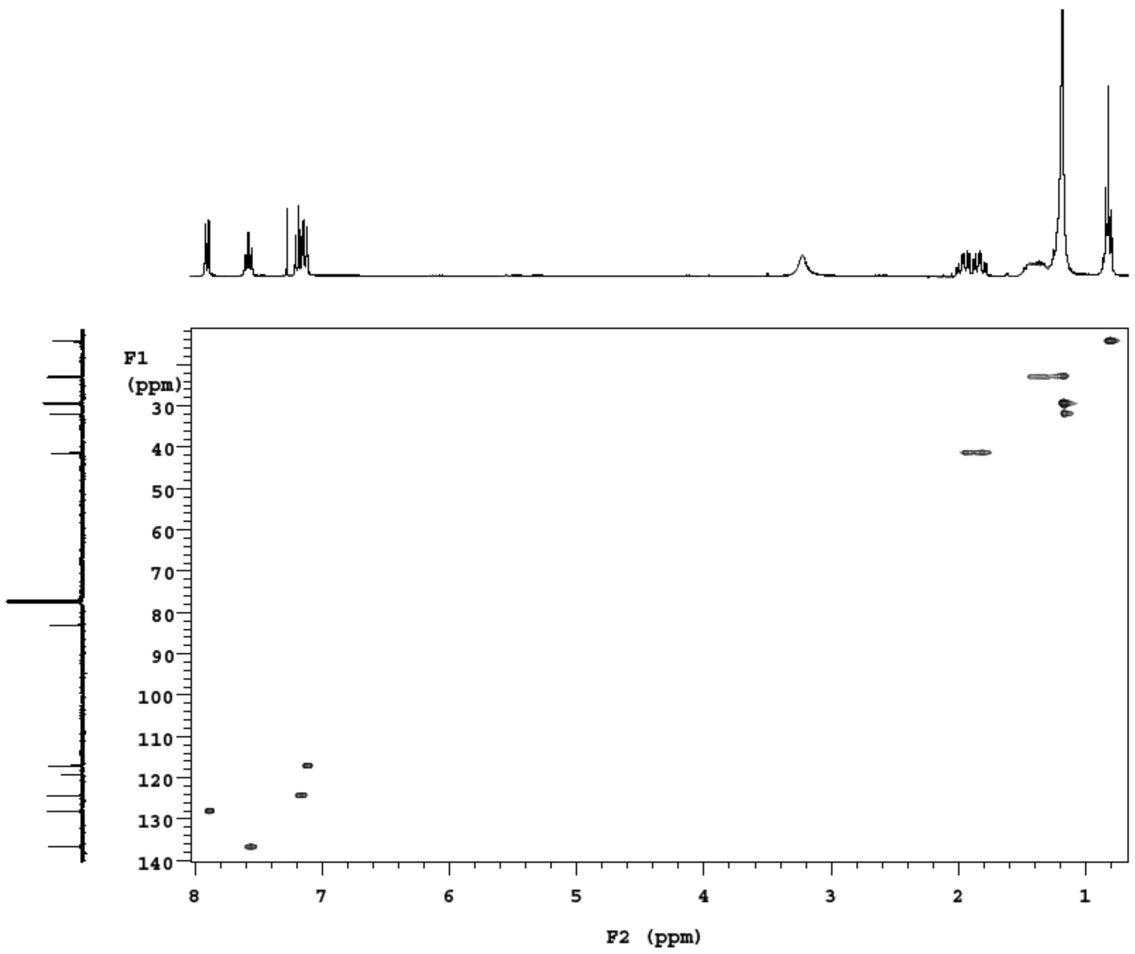

Figure S4. HMQC spectrum of compound **1** (300 MHz, CD_3OD)

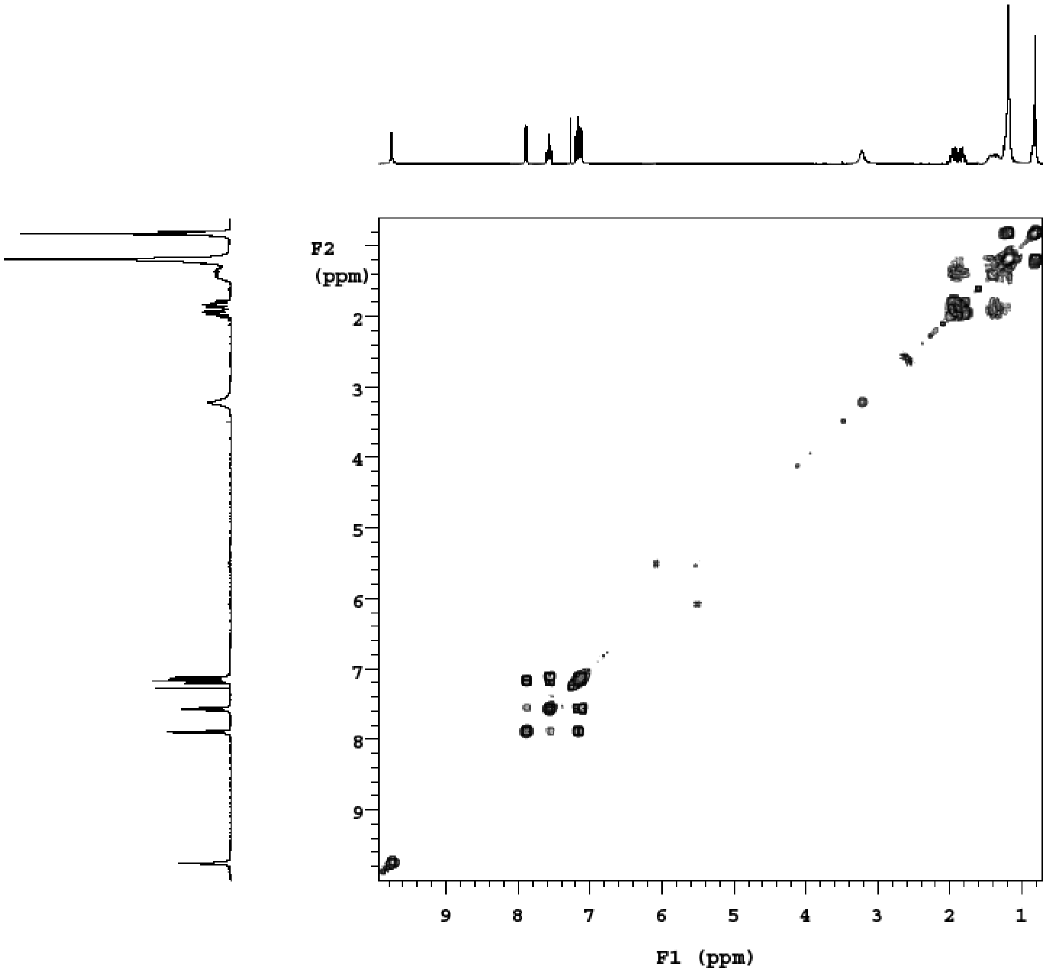

Figure S5. COSY spectrum of compound **1** (300 MHz, CD_3OD)

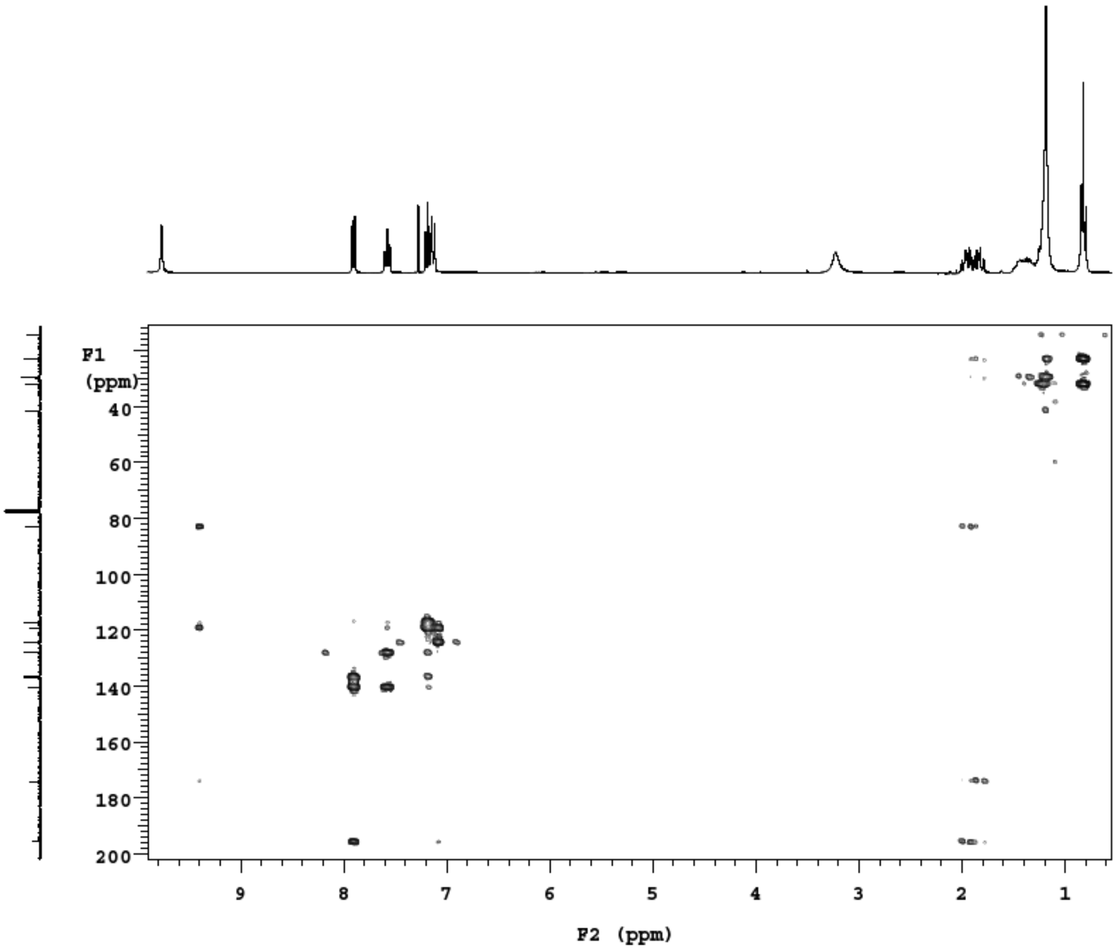

Figure S6. HMBC spectrum of compound **1** (300 MHz, CD_3OD)

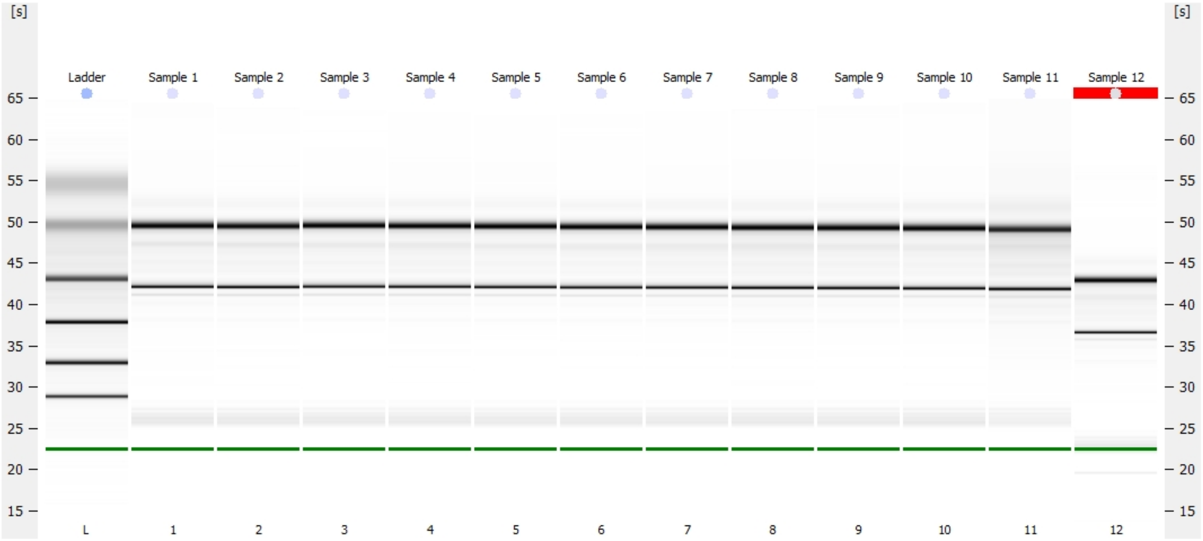

Figure S7. Electropherogram of RNA samples obtained from 24h treatments.

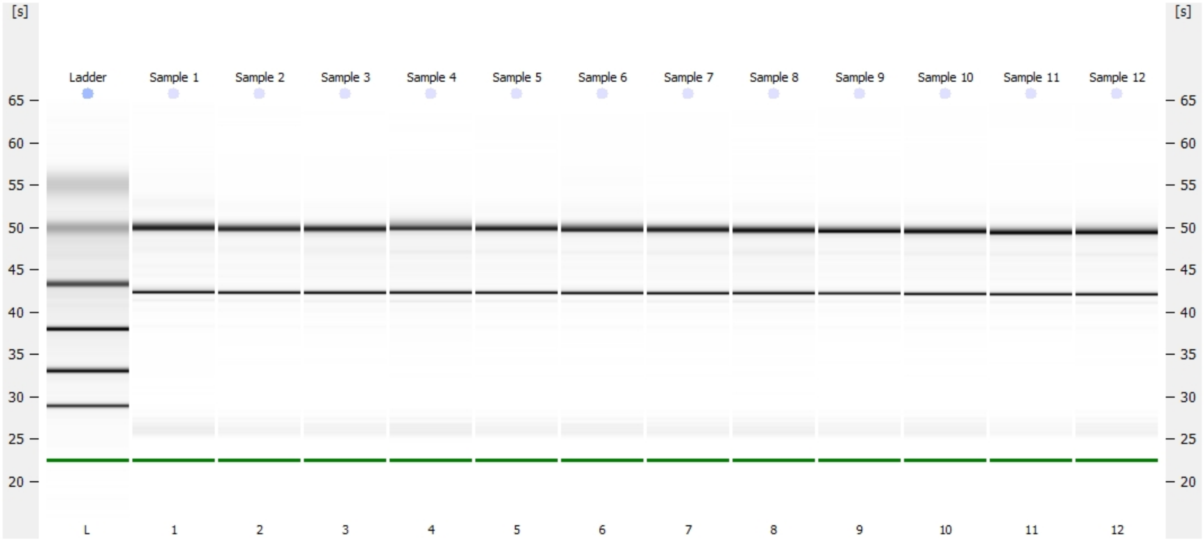

Figure S8. Electropherogram of RNA samples obtained from 48h treatments.
