# Supplementary material for: RNAm expression profile of cancer marker genes in HepG2 cells treated with different concentrations of a new indolin-3-one from Pseudomonas aeruginosa
Source: Sci Rep. 2018 Aug 24;8:12781. doi: 10.1038/s41598-018-30893-w (PMC6109079; doi:10.1038/s41598-018-30893-w)
Supplement: Supplementary file 2 — Supplementary Tables [file 41598_2018_30893_MOESM2_ESM.pdf]

**Gene/indolin-3-one concentration** (μM)	**Expression**	**Std. Error**	**95% C.I.**	**P(H1)**	**Result *p*<0.05**
MET IND 20	2.405	0.581 - 7.191	0.360 - 12.174	0.045	UP
MET IND 40	0.948	0.474 - 1.814	0.283 - 3.031	0.821	
MET IND 60	0.54	0.310 - 0.881	0.202 - 1.405	0.006	DOWN
MYC IND 20	1.684	1.016 - 2.846	0.692 - 3.736	0.009	UP
MYC IND 40	0.756	0.324 - 1.481	0.216 - 1.816	0.257	
MYC IND 60	0.386	0.201 - 0.642	0.142 - 0.786	0	DOWN
CDK2 IND 20	2.058	0.871 - 4.954	0.417 - 6.742	0.03	UP
CDK2 IND 40	1.103	0.640 - 1.626	0.479 - 2.197	0.524	
CDK2 IND 60	0.572	0.316 - 1.024	0.161 - 1.528	0.019	DOWN
CDK4 IND 20	1.042	0.438 - 2.500	0.196 - 4.193	0.881	
CDK4 IND 40	0.686	0.317 - 1.367	0.161 - 1.773	0.123	
CDK4 IND 60	0.558	0.245 - 1.255	0.094 - 2.933	0.07	
CDK6 IND 20	1.439	0.624 - 3.163	0.325 - 4.209	0.203	
CDK6 IND 40	0.681	0.417 - 1.052	0.312 - 1.301	0.02	DOWN
CDK6 IND 60	0.352	0.205 - 0.583	0.145 - 0.848	0.0	DOWN
CDKN1A IND 20	1.745	1.059 - 2.907	0.712 - 3.474	0.006	UP
CDKN1A IND 40	1.023	0.643 - 1.591	0.394 - 2.057	0.887	
CDKN1A IND 60	0.688	0.406 - 1.158	0.283 - 1.828	0.057	
CDKN2A IND 20	1.762	1.186 - 2.705	0.823 - 3.496	0.003	UP
CDKN2A IND 40	1.118	0.729 - 1.732	0.534 - 2.987	0.493	
CDKN2A IND 60	0.876	0.473 - 1.491	0.380 - 2.127	0.457	
CCND1 IND 20	1.554	0.692 - 2.840	0.522 - 3.714	0.07	
CCND1 IND 40	0.772	0.583 - 1.055	0.507 - 1.338	0.018	DOWN
CCND1 IND 60	0.4	0.284 - 0.556	0.245 - 0.808	0	DOWN
CCNA2 IND 20	1.548	0.962 - 2.370	0.690 - 3.511	0.016	UP
CCNA2 IND 40	1.029	0.678 - 1.498	0.501 - 2.117	0.839	
CCNA2 IND 60	0.626	0.339 - 1.108	0.219 - 1.659	0.034	DOWN
EG5 IND 20	2.624	0.696 - 8.230	0.323 - 10.564	0.027	UP
EG5 IND 40	1.033	0.549 - 2.084	0.239 - 2.789	0.894	
EG5 IND 60	0.836	0.493 - 1.408	0.305 - 1.755	0.32	
EIF IND 20	2.719	0.528 - 10.862	0.285 - 16.198	0.038	UP
EIF IND 40	1.253	0.545 - 2.791	0.462 - 4.916	0.379	

EIF IND 60	0.664	0.276 - 1.595	0.200 - 2.978	0.154	
E2F IND 20	1.461	0.924 - 2.177	0.755 - 2.912	0.013	UP
E2F IND 40	0.767	0.539 - 1.063	0.476 - 1.482	0.03	DOWN
E2F IND 60	0.429	0.325 - 0.569	0.257 - 0.741	0	DOWN
BIRC5 IND 20	1.519	1.035 - 2.396	0.553 - 3.375	0.024	UP
BIRC5 IND 40	1.058	0.660 - 1.760	0.412 - 2.425	0.759	
BIRC5 IND 60	0.632	0.432 - 1.053	0.242 - 1.253	0.005	DOWN
TP53 IND 20	1.397	0.573 - 2.746	0.342 - 3.231	0.18	
TP53 IND 40	0.592	0.386 - 0.930	0.232 - 1.337	0.01	DOWN
TP53 IND 60	0.276	0.167 - 0.505	0.103 - 0.759	0	DOWN

Table S1. 24 hours' data analysis of HepG2 gene expression using the standalone software REST 2009, with efficiency correction. All expression levels, standard errors, 95% confidence index and *p* values are described.

Gene/indolin-3-one concentration (μM)	Expression	Std. Error	95% C.I.	P(H1)	Result $p<0.05$
MET IND 20	0.864	0.589 - 1.236	0.507 - 1.764	0.226	
MET IND 40	0.752	0.498 - 1.110	0.398 - 1.613	0.057	
MET IND 60	0.417	0.279 - 0.626	0.191 - 0.861	0	DOWN
MYC IND 20	0.998	0.640 - 1.659	0.562 - 2.372	0.993	
MYC IND 40	0.905	0.482 - 1.325	0.428 - 2.397	0.56	
MYC IND 60	0.685	0.540 - 0.832	0.445 - 1.348	0.002	DOWN
CDK2 IND 20	0.793	0.532 - 1.119	0.433 - 2.167	0.142	
CDK2 IND 40	0.659	0.458 - 0.994	0.350 - 1.232	0.006	DOWN
CDK2 IND 60	0.556	0.389 - 0.774	0.301 - 1.421	0.001	DOWN
CDK4 IND 20	0.781	0.460 - 1.298	0.249 - 1.817	0.205	
CDK4 IND 40	0.647	0.340 - 1.049	0.227 - 1.372	0.029	DOWN
CDK4 IND 60	0.558	0.302 - 0.927	0.189 - 1.275	0.003	DOWN
CDK6 IND 20	0.973	0.653 - 1.473	0.358 - 1.821	0.883	
CDK6 IND 40	0.822	0.428 - 1.304	0.364 - 1.528	0.251	
CDK6 IND 60	0.732	0.461 - 1.203	0.332 - 1.581	0.063	
CDKN1A IND 20	0.99	0.483 - 1.655	0.362 - 2.056	0.95	
CDKN1A IND 40	0.91	0.534 - 1.311	0.427 - 1.857	0.539	
CDKN1A IND 60	0.903	0.580 - 1.491	0.465 - 2.203	0.51	
CDKN2A IND 20	1.001	0.768 - 1.281	0.607 - 1.535	0.994	
CDKN2A IND 40	0.985	0.742 - 1.548	0.615 - 1.950	0.894	
CDKN2A IND 60	0.854	0.703 - 1.054	0.521 - 1.290	0.064	
CCND1 IND 20	0.948	0.586 - 1.426	0.455 - 2.205	0.715	
CCND1 IND 40	0.64	0.506 - 0.916	0.330 - 1.281	0.002	DOWN
CCND1 IND 60	0.418	0.298 - 0.592	0.245 - 0.884	0	DOWN
CCNA2 IND 20	0.722	0.493 - 1.073	0.373 - 1.343	0.017	DOWN
CCNA2 IND 40	0.675	0.419 - 1.041	0.254 - 1.620	0.029	DOWN
CCNA2 IND 60	0.573	0.377 - 0.863	0.283 - 1.192	0.001	DOWN
EG5 IND 20	0.541	0.252 - 1.131	0.148 - 1.404	0.024	DOWN
EG5 IND 40	0.485	0.247 - 0.930	0.153 - 1.165	0.002	DOWN
EG5 IND 60	0.413	0.274 - 0.642	0.177 - 0.802	0	DOWN
EIF IND 20	1.483	0.816 - 2.496	0.546 - 4.320	0.059	

EIF IND 40	0.952	0.354 - 2.434	0.175 - 3.428	0.869	
EIF IND 60	0.862	0.396 - 1.803	0.249 - 3.766	0.59	
E2F IND 20	1.697	1.166 - 2.589	0.879 - 2.900	0.001	UP
E2F IND 40	1.945	1.271 - 2.890	0.840 - 3.540	0.001	UP
E2F IND 60	1.233	0.820 - 1.958	0.589 - 2.727	0.167	
BIRC5 IND 20	0.982	0.663 - 1.384	0.531 - 1.653	0.883	
BIRC5 IND 40	0.887	0.592 - 1.338	0.454 - 1.690	0.386	
BIRC5 IND 60	0.461	0.309 - 0.741	0.254 - 0.983	0	DOWN
TP53 IND 20	1.105	0.897 - 1.394	0.711 - 1.642	0.229	
TP53 IND 40	0.89	0.694 - 1.214	0.578 - 1.338	0.193	
TP53 IND 60	0.663	0.517 - 0.861	0.417 - 1.071	0.001	DOWN

Table S2. 48 hours' data analysis of HepG2 gene expression using the standalone software REST 2009, with efficiency correction. All expression levels, standard errors, 95% confidence index and *p* values are described.
